# Creating a Novel Origin of Replication through Modulating DNA-Protein Interfaces

**DOI:** 10.1371/journal.pone.0008850

**Published:** 2010-01-22

**Authors:** F. Curtis Hewitt, R. Jude Samulski

**Affiliations:** 1 Gene Therapy Center, University of North Carolina at Chapel Hill, Chapel Hill, North Carolina, United States of America; 2 Curriculum in Genetics and Molecular Biology, University of North Carolina at Chapel Hill, Chapel Hill, North Carolina, United States of America; The University of Chicago, United States of America

## Abstract

**Background:**

While the molecular mechanisms of DNA-protein specificity at the origin of replication have been determined in many model organisms, these interactions remain unknown in the majority of higher eukaryotes and numerous vertebrate viruses. Similar to many viral origins of replication, adeno-associated virus (AAV) utilizes a *cis*-acting origin of replication and a virus specific Replication protein (Rep) to faithfully carry out self-priming replication. The mechanisms of AAV DNA replication are generally well understood. However, the molecular basis of specificity between the Rep protein and the viral origin of replication between different AAV serotypes remains uncharacterized.

**Methodology/Principal Findings:**

By generating a panel of chimeric and mutant origins between two AAV serotypes, we have mapped two independent DNA-Protein interfaces involved in replicative specificity. In vivo replication assays and structural modeling demonstrated that three residues in the AAV2 Rep active site are necessary to cleave its cognate origin. An analogous origin (AAV5) possesses a unique interaction between an extended Rep binding element and a 49 aa region of Rep containing two DNA binding interfaces.

**Conclusions/Significance:**

The elucidation of these structure-function relationships at the AAV origin led to the creation of a unique recombinant origin and compatible Rep protein with properties independent of either parent serotype. This novel origin may impact the safety and efficacy of AAV as a gene delivery tool. This work may also explain the unique ability of certain AAV serotypes to achieve site-directed integration into the human chromosome. Finally, this result impacts the study of conserved DNA viruses which employ rolling circle mechanisms of replication.

## Introduction

General understanding of the mechanisms required for function at origins of replication has grown immensely since the first prokaryotic origins were characterized. While the DNA-protein interactions necessary for replication in prokaryotes, lower eukaryotes, and bacteriophages are generally well understood, mechanisms employed in the majority of higher eukaryotes and vertebrate viruses, such as adeno-associated virus (AAV), are still being determined. The inverted terminal repeats (ITRs) of AAV and other Parvoviruses act as the origin of replication. These elements flank the short, single stranded genome and typically possess a T-shaped secondary structure. The replication strategies of the genus *Dependovirus*, specifically those of adeno-associated virus (AAV), have been well characterized. The viral non-structural or Replication proteins (Rep) are the only factors required to interact with the ITR in order to catalyze replication [1]. The majority of AAV serotypes possess highly conserved origins of replication with interchangeable DNA-protein interactions. However, the Rep proteins of several serotypes interact exclusively with their cognate ITR. Discovering the mechanisms which drive Rep-ITR specificity promises to advance our understanding of DNA-protein interactions at viral origins of replication. These findings also promise to shed light on how eukaryotic and prokaryotic proteins achieve selectivity to DNA substrates.

**Figure 1:**
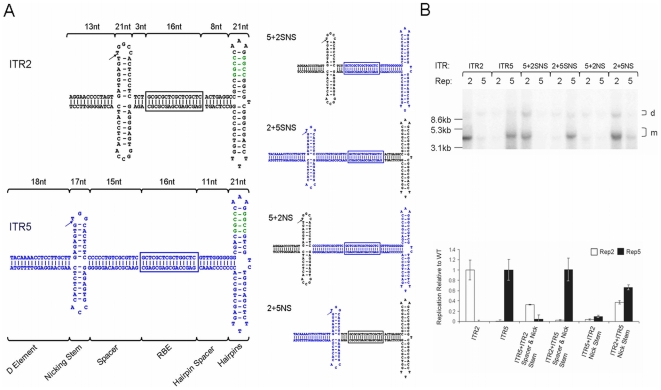
Cloning and characterization of chimeric ITRs. (A) Sequence and structure of ITR2 (black) and ITR5 (blue) shown with incorporation of *SfiI* sites for cloning (green). Length in nt of ITR elements indicated above brackets. RBE is boxed. RBE' is indicated by a hatched circle. Nicking stem is extruded with arrow indicating the nicking site and hatched box indicating the trs. The four initial chimeric ITRs generated are shown (right). (B) Replication assay and quantitation of chimeric Reps. Replication products from the indicated ITR and either Rep2 or Rep5 were analyzed by Southern blot. Monomeric (m) and dimeric (d) replicating species are indicated. The level of replication of each sample was measured by densitometric analysis and compared to wt replication.

The AAV ITRs are critical for nearly every aspect of the viral life-cycle (Fig. S1). The secondary structure of the ITR is necessary to prime synthesis of the second strand to allow transcription of the viral genes [2]. The full length Rep proteins contain a unique N-terminal DNA binding region which specifically recognizes the ITR at the 16 nt Rep binding element (RBE) and at the tip of one of the hairpin stems known as the RBE' (Fig. 1A) [3], [4]. Rep molecules multimerize on the ITR allowing the C-terminus of Rep, acting as an ATP-dependent SF3 helicase, to unwind the ITR and form a putative internal hairpin [1], [5]. This hairpin, (here, termed ‘nicking stem’) contains the terminal resolution site (trs) where Rep nicks the ITR in a site-specific manner (Fig. 1A) [6]. This DNA cleavage is required for replication of the closed ITR and is necessary to initiate subsequent rounds of genomic replication. Replicated genomes can undergo replication again or be encapsidated in the presence of the smaller Rep proteins [7].

The ITR sequences of seven human/primate AAV serotypes have been published. These sequences typically display 80% or greater nucleotide conservation and segregate into two groups [8]. The AAV2 Rep proteins (Rep2) are able to function on the ITR of nearly every known AAV serotype except those of AAV5 (ITR5) [8], [9]. Consistently, Rep5 is unable to catalyze replication of ITR2 (Fig. 1B). Replicative specificity between these serotypes does not exist at the level of binding, as Rep2 and Rep5 can bind interchangeably to ITR2 or ITR5 [10]. Instead, specificity is created by the inability of Rep to cleave the ITR of the opposite serotype. This occurs despite high conservation between the ITR2 and ITR5 sequence, secondary structure, and location of elements required for Rep interaction (RBE, RBE', trs, nicking stem, Fig. 1A).

Identification of the elements involved in Rep-ITR specificity stands to increase the understanding of viral and cellular DNA binding and endonucleolytic proteins. It is likely that similar interactions take place in a wide range of viral and cellular replication and repair pathways. Localization of these elements may also facilitate the identification of other unique Parvovirus origins of replication. Here, we demonstrate two unique mechanisms at the DNA and protein level to achieve Rep-ITR specificity and utilize these factors to create a novel AAV origin of replication.

## Results

### Construction and Characterization of Chimeric ITRs

Previously, AAV replicative specificity was postulated to be driven by the trs sequence [10], [11]. Rep2 can nick the ITR2 trs (AGT/TGG) and the AAVS1 trs of human chromosome 19 (GGT/TGG) [12]. Rep5 nicks only the ITR5 trs (AGTG/TGG). However, alignment of the ITR2 and ITR5 sequences revealed several significant sequence and structural differences outside the trs sequence (Fig. 1A). The spacing between the putative RBE and the nicking stem was significantly different; three nucleotides (nt) for ITR2 and 15 nt for ITR5. Additionally, while the trs sequence is not tightly conserved between ITR2 and ITR5, neither is the height or overall length of the putative nicking stem.

Here, we utilized a novel method to generate mutant ITRs in order to determine which portions of the ITR were responsible for replicative specificity. Previous studies have investigated Rep-ITR interactions *in vitro* largely due to the difficulty of synthesizing full length ITRs for *in vivo* assays. PCR through the secondary structure of the ITR is inefficient and sequencing through these elements typically requires radiolabeled chain-terminator sequencing [13]. The AAV ITRs are highly recombinogenic and are frequently mutated even in a plasmid context [14].

In order to address these concerns the ITR was synthesized and amplified in halves (Fig. S2). Assembly of the halves required the inclusion of a *SfiI* site in one of the hairpin arms of the ITR. *SfiI* allowed the conservation of the RBE' sequence [4]. Cloning the ITR in a DD format required only one ITR per plasmid for replication [15]. The three core Rep functions necessary for AAV replication (Rep binding, helicase, and nicking) were analyzed by the presence or absence of intracellular replication of the plasmid. This assay provided the ability to quantitate Rep-ITR function in a physiological setting, removing the concern that highly purified Rep protein might take on aberrant function *in vitro*. This system also avoided concerns that previous *in vitro* assays used only a fragment of the ITR or that oligos used to recapitulate the ITR might not fold correctly.

An alignment of ITR2 and ITR5 (Fig. 1A) revealed several divergent elements which might infer Rep specificity. The RBE and hairpins seemed unlikely to impact specificity as Rep2 and Rep5 have been reported to bind ITR2 and ITR5 interchangeably [10]. Additionally, no evidence of Rep interactions with the portion of the D-element outside the nicking stem has been presented. Therefore, the spacer and nicking stem elements appeared to be the most likely candidates for unique interactions with their cognate Rep protein. This hypothesis was supported by low homology of these elements between AAV2 and AAV5.

Wt ITRs containing the *SfiI* site functioned as expected with Rep2 specific to ITR2 and Rep5 specific to ITR5 (Fig. 1B). Rep2-ITR2 replicated approximately 2-fold better than Rep5-ITR5, potentially due to the lower folding energy of ITR5 resulting in reduced plasmid stability prior to replication. Due to this minor difference in replicative fidelity, all ITRs replicated with Rep2 were normalized to Rep2-ITR2, while ITRs replicated with Rep5 were normalized to Rep5-ITR5 (Fig. 1B).

In order to confirm that the RBE and hairpin arms played no role in Rep specificity, we generated a chimeric ITR with ITR5 binding elements and an ITR2 spacer and nicking stem (ITR5+2SNS). Only Rep2 replicated this ITR, confirming the determinants of replicative specificity lie in the spacer/nicking stem elements (Fig. 1B). While ITR5+2SNS replication was not as efficient as ITR2-Rep2, it was replicated at ITR5-Rep5 levels. Conversely, Rep5 specifically replicated an ITR comprised of ITR2 hairpins and hairpin spacer and the ITR5 spacer and nicking stem (ITR2+5SNS, Fig. 1B). Rep5 replicated this ITR at wt levels. This data demonstrated that Rep-ITR specificity lies outside of the ITR binding regions.

Next, we explored whether the nicking stem or the spacing between the RBE and nicking stem harbored unique interactions with the Rep protein by creating chimeric ITRs which divided these individual elements. An ITR with the ITR5 binding elements and spacer and the ITR2 nicking stem could not be replicated by either Rep2 or Rep5 (ITR5+2NS, Fig. 1B). The corresponding chimeric ITR (ITR2 binding elements and spacer with an ITR5 nicking stem) was replicated by both Rep2 and Rep5 (ITR2+5NS, Fig. 1B). This disparity suggested that the spacer and nicking stem play different roles in Rep-ITR specificity between AAV2 and AAV5.

### The Nicking Stem Is Critical for ITR5 Specificity

**Figure 2:**
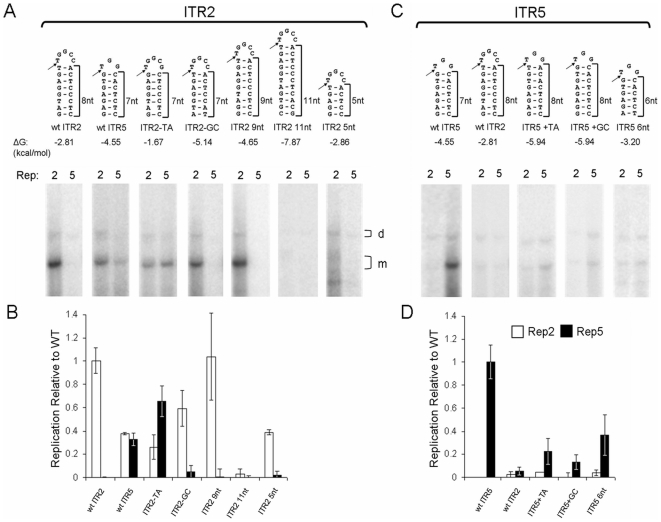
Relation of nicking stem height and sequence to Rep-ITR specificity. (A) Sequence of nicking stem in an otherwise ITR2 context. Arrow indicates trs site. Brackets indicate height of putative stems in nt from the base of the stem to the putative nicking site. Predicted ΔG values for the hairpins are below. Southern blot analysis of the ITRs replicated by Rep2 or Rep5 are shown below. (B) Quantitation of the Southern blots relative to wt replication from (A). (C) Same as (A), except nicking stems indicated were used in an ITR5 context. (D) Quantitation of the Southern blots relative to wt replication from (C).

ITR2+5NS established that Rep2 is capable of nicking an ITR with an ITR5 nicking stem and that Rep-ITR specificity is not driven exclusively by the trs sequence (Fig. 1B). In order to determine the flexibility of Rep2 toward mutant nicking stems, we generated ITR2s containing altered forms of the hairpin (Fig. 2A). Rep2 is able to replicate an ITR with an ITR5 nicking stem even though the ITR5 nicking stem contains a different trs sequence, is one bp shorter, and has two fewer unpaired nucleotides at its tip (Fig. 2A). The substitution of the ITR5 nicking stem into ITR2 also allowed replication by Rep5.

To determine which element of the ITR2 nicking stem prevented Rep5 activity, we altered specific portions of the ITR2 stem. First, one bp at the top of the putative ITR2 nicking stem was removed to lower the height to that of ITR5 (ITR2-TA). Removing the T-A bp also resulted in a trs resembling ITR5, nicking between G/T opposed to T/T. Rep2 continued to function on this ITR as did Rep5, demonstrating that Rep5 can tolerate five unpaired nucleotides at the tip of the stem as long as the stem height and nt sequence are correct. A similar deletion from the base of the ITR2 nicking stem reduced the height to that of ITR5 while retaining the ITR2 nicking site (ITR2-GC). Rep2 continued to function efficiently on this ITR while Rep5 activity was ablated. This data suggested that the inability of Rep5 to function on ITR2 is primarily the sequence of the trs, specifically the requirement for a nick to be generated between G/T.

To determine the extent of Rep2 flexibility for different nicking stems, we created three additional ITR2 mutants. Extending the nicking stem by one bp at the base had no effect on replication by Rep2 (ITR2 9 nt). However, a three bp extension was sufficient to ablate Rep2 function on the ITR (ITR2 11 nt). Surprisingly, Rep2 was able to tolerate a three bp deletion from the base of the stem, underlining the flexibility of Rep2 with respect to nicking stem substrates (ITR2 5 nt).

In order to explore the level of flexibility Rep5 possessed toward non-wt nicking stems, we created a panel of mutant ITR5s harboring altered nicking stems (Fig. 2C). Curiously, Rep2 replicated none of these ITRs, suggesting an element outside the ITR5 nicking stem is responsible for preventing Rep2 function. As in Figure 1B, replacement of the ITR5 nicking stem with that of ITR2 resulted in the ablation of replication by Rep5, attributable to the incompatible trs sequence. The addition of one bp at the top of the ITR5 nicking stem severely decreased the ability of Rep5 to replicate the ITR (ITR5 +TA, Fig. 2D). This insertion disrupted the ITR5 trs sequence and increased the size of the stem one bp. However, the low level of replication by Rep5 on ITR5 +TA suggests that the entire trs site of ITR2 is necessary to confer Rep2 specificity, not just the presence of a T/T nick site.

The addition of one bp to the base of the ITR5 nicking stem, preserving the ITR5 trs at the tip, nearly eliminated replication by Rep5 (ITR5 +GC). Likewise, the removal of one bp from the base of the ITR5 nicking stem strongly decreased replication by Rep5 (ITR5 6nt, Fig. 2D). This data suggests that Rep5 is sensitive both to the height of the nicking stem as well as to the sequence of the trs. Thus, Rep5 is unable to replicate ITR2 because the ITR2 nicking stem is one bp too tall and has an incompatible trs sequence.

### Spacer Length Is Critical for ITR2, Not ITR5

While Rep2 can replicate a vector with an ITR5 nicking stem, it can not replicate wt ITR5 (Fig. 1B). The only difference between ITR5+2SNS (which Rep2 can replicate) and ITR5+2NS (which Rep2 can not) is the ITR5 spacer (Fig. 1B). The wt Rep2 spacer is three nt long while the wt Rep5 spacer is 15 nt long. Thus, we hypothesized that Rep2 may be sensitive to spacer length. Previous *in vitro* data supported this conclusion as insertions into the ITR2 spacer prevented nicking by Rep2 [4].

**Figure 3:**
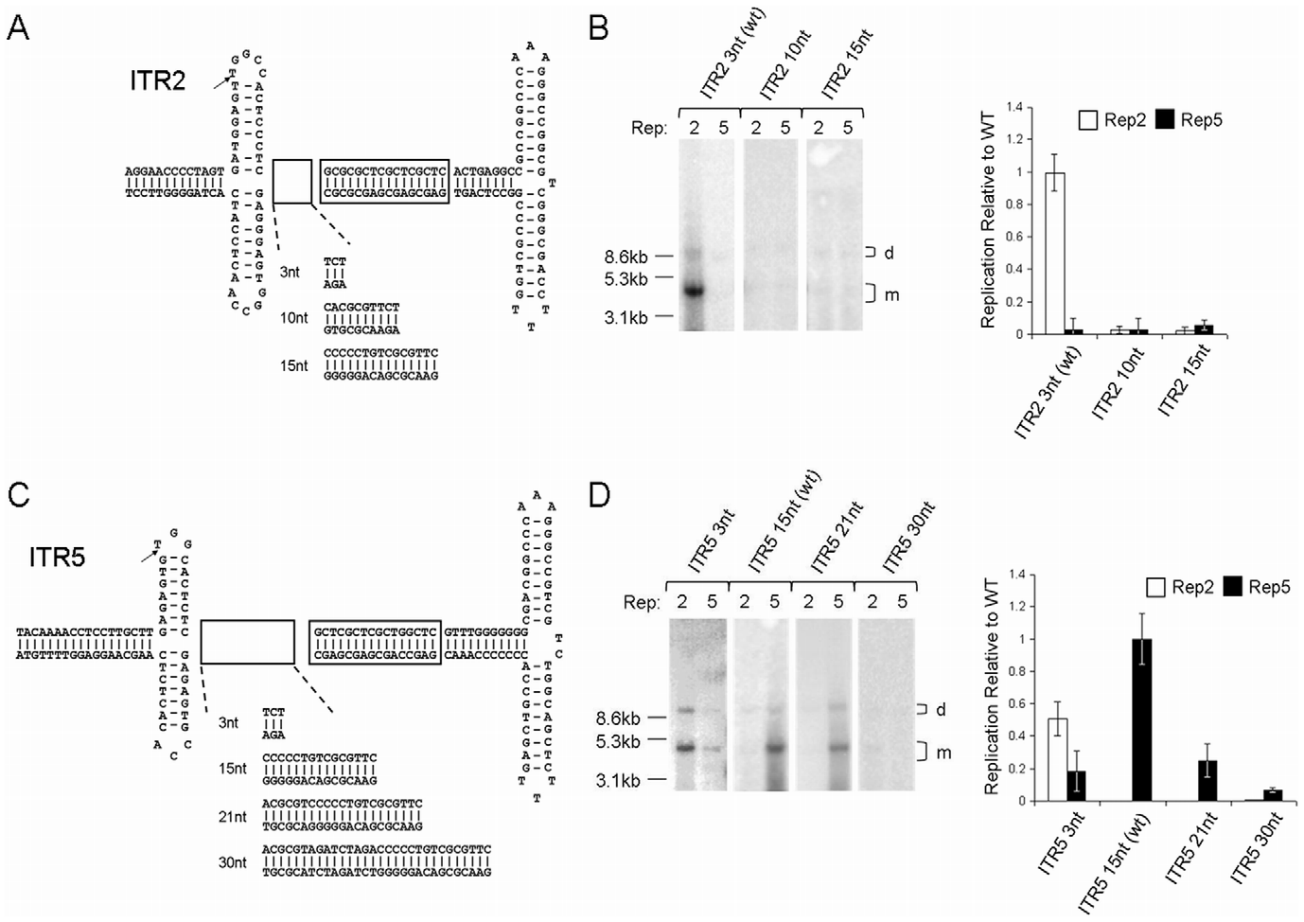
Effect of RBE-nicking stem spacing on Rep-ITR specificity. (A) ITR2 mutants were synthesized with the indicated spacing between the RBE and nicking stem. (B) Southern blot analysis of the ITRs depicted in (A) replicated by either Rep2 or Rep5 (Left). Quantitation of Southern blots relative to wt replication (Right). (C) ITR5 mutants synthesized as in (A). (D) Southern blot analysis and quantitation of (C).

To explore the effect of spacer length on ITR2 and ITR5, we generated a series of mutant ITR2s and ITR5s with differing spacer lengths (Fig. 3A and 3C). An insertion extending the ITR2 spacer to 10 nt ablated replication by Rep2 (ITR2 10nt, Fig. 3B). Similarly, substitution of the ITR2 spacer with the 15 nt spacer of ITR5 also ablated replication by Rep2 (ITR2 15nt, Fig. 3B). Rep5 was unable to replicate any of these vectors due to the presence of the ITR2 stem loop.

Rep5 displayed greater flexibility toward spacer elements of differing lengths. Replacing the 15 nt ITR5 spacer with that of ITR2 resulted in an ITR which Rep5 retained the ability to replicate at a reduced level (ITR5 3nt, Fig. 3D). Additionally, the presence of the three nt spacer allowed Rep2 to function on this ITR. The addition of six nt to the ITR5 spacer (for a total spacer length of 21nt) resulted in an ITR capable of being replicated by Rep5 at an efficient level (ITR5 21nt, Fig. 3D). Replication by Rep5 was effectively abolished only after the insertion of 15 nt into the spacer (ITR5 30nt, Fig. 3D). This panel of mutant ITR5s demonstrates the requirement for a three nt spacer element for Rep2 function.

This data confirmed that the length of the ITR5 spacer was critical to block Rep2 function. Even small insertions into the ITR2 spacer were not tolerated by Rep2. Meanwhile, Rep5 is flexible in regard to spacer length, demonstrating the ability to function on ITRs with spacers from 3–21 nt.

### The ITR5 Spacer Acts as a RBE for Rep5

**Figure 4:**
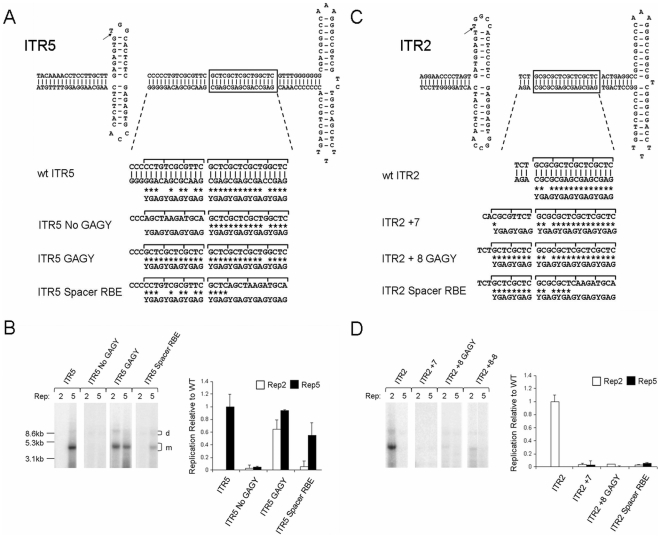
The ITR5 spacer acts as a RBE for Rep5. (A) ITR5 mutants were synthesized with the indicated RBE and spacer sequence. Brackets indicate individual tetranucleotide repeats bound by Rep monomers. Both strands of the wt ITR5 sequence are shown to illustrate conservation with the GAGY motif (indicated by *). Only one strand shown on others. (B) Southern blot analysis of the ITRs depicted in (A) replicated by either Rep2 or Rep5 (Left). Quantitation of Southern blots relative to wt replication (Right). (C) ITR2 mutants were generated with the RBE and spacer sequences indicated. (D) Southern blot analysis and quantitation for (C).

The inability of Rep2 to function on ITRs with spacers longer than three nt led to the question of why Rep5 was so flexible in this regard. We hypothesized that Rep5 might specifically bind the ITR5 spacer just as it binds the RBE. The inability of Rep2 to bind this sequence would preclude its function on ITR5. Supporting this hypothesis was a moderately conserved GAGY Rep binding motif extending throughout the ITR5 spacer (Fig. 4A). Additionally, as Rep monomers bind every four nt, the binding of three Rep5 monomers to the 15 nt spacer element would result in a three nt spacer, similar to that of ITR2 [16].

If Rep5 does bind the loosely conserved GAGY motif, the removal of that motif from the spacer should abolish Rep5 function. Indeed, the ITR5 No GAGY mutant could not be replicated by Rep2 or Rep5 (Fig. 4B). This suggested that the specific sequence of the ITR5 spacer plays an active role in the Rep5-ITR5 interaction. Conversely, a spacer with a pure GAGY repeat should not disrupt the ability of Rep5 to function on the ITR. Indeed, Rep5 was able to replicate this ITR at wt levels (ITR5 GAGY, Fig. 4B). Rep2 was also able to replicate this ITR efficiently, suggesting the poorly conserved nature of the GAGY repeat within the ITR5 spacer prevents a critical DNA-protein interaction with Rep2 necessary for replication.

To explore how the ITR5 spacer functioned as an RBE, we removed three GAGY repeats from the hairpin side of the RBE (ITR5 Spacer RBE, Fig. 4A). This essentially shifted the 16 nt RBE 12 nt closer to the nicking stem. Rep5 replicated this ITR efficiently, confirming the ITR5 spacer acts as a RBE (ITR5 Spacer RBE, Fig. 4B). The slight reduction in replication fidelity of this ITR with respect to wt ITR5 may signal the inability Rep to properly interact with the RBE' [4]. Rep2 was again unable to replicate ITR5 Spacer RBE due to its inability to interact with the ITR5 spacer.

Next, we sought to extend the ITR2 spacer element to function as an extended RBE (Fig. 4C). The seven nt insertion attempted in Figure 3A possessed essentially no GAGY homology (ITR2 +7, Fig. 4C). As a result, Rep2 could not replicate this ITR (Fig. 4D). Eight nt (two four nt GAGY repeats) inserted into the ITR2 spacer between the RBE and the existing spacer prevented replication by Rep2, demonstrating that the ITR2 RBE can not be extended. This suggests that Rep2 may be dependent on RBE' binding or a specific spacer length critical for proper oligomerization to function on its cognate ITR. Curiously, this requirement does not apply to Rep2 function on ITR5 GAGY (Fig. 4A).

Similar to ITR5 Spacer RBE, we retained the eight nt GAGY insertion into ITR2 while removing eight nt of GAGY from the hairpin side of the RBE (ITR2 +8 −8, Fig. 4C). This shifted the RBE eight nt closer to the nicking stem. Rep2 replicated this ITR very inefficiently at a level below the detection threshold of densitometric analysis (Fig. 4D, Southern).

### Identification of Regions in Rep Responsible for ITR Specificity

**Figure 5:**
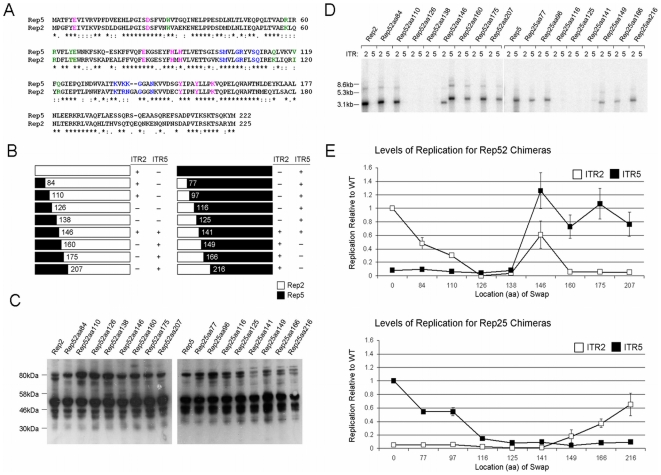
Cloning and characterization of chimeric Reps. (A) An alignment of the N-termini of Rep2 and Rep5. (*) represents conserved amino acids. (: and.) indicates conservative substitutions. Blue indicates residues implicated in RBE binding interactions. Pink indicates residues which participate in the endonucleolytic active site. Green indicates residues implicated in RBE' binding. (B) Chimeric Reps created and their ability to replicate ITR2 or ITR5 flanked vectors. Numbers indicate the aa position of the switch from one Rep to the other. (+) indicates the presence of replication, (−) indicates the absence. (C) Western blot for expression of the chimeric Reps. (D) Southern blot demonstrating replication of an ITR2 or an ITR5 vector by the chimeric Reps. Note that the ITR5 vector is 500bp larger than the ITR2 vector. (E) Level of replication of the chimeric Reps relative to wt Rep2 or Rep5.

Identifying the two elements of the ITR responsible for Rep specificity allowed us to map the regions of Rep2 and Rep5 involved in ITR specificity. We focused exclusively on the N-terminal 208 aa of the large Rep proteins as this region encompasses the DNA binding and endonucleolytic activity of the protein [17]. This region displays approximately 60% sequence conservation evenly distributed across the protein sequence (Fig. 5A). Residues involved in the active site of the protein are 100% conserved between Rep2 and Rep5 [18]. Residues implicated in binding the RBE' are highly conserved [16]. Residues which bind the RBE display nearly perfect conservation except for two conservative substitutions near aa 140.

In order to map the regions of Rep involved in ITR specificity, we generated a panel of chimeric Reps derived from Rep2 and Rep5 (Fig. 5B). The ability of each chimeric Rep to replicate an ITR2- or ITR5-flanked vector in HEK 293 cells was determined by Southern blot (Fig. 5B and 5D). Each Rep in the panel was verified by DNA sequencing and Western blot analysis (Fig. 5C). Every chimeric Rep showed similar protein expression profiles compared to wt. Densitometric analysis provided a comparison of the replication efficiency of each chimeric Rep with that of wt Rep2 or Rep5 (Fig. 5E). Chimeric Reps were named according to the aa location of the swap between serotypes; for instance, Rep25aa77 possesses the N-terminal 76 aa of Rep2 and the C-terminus of Rep5.

In the case of Rep5, replacement of the N-terminal 77 or 97 aa with Rep2 had no effect on ITR specificity nor a significant impact on replicative fidelity (Fig. 5D and 5E). Larger pieces of Rep2 substituted onto the N-terminus of Rep5 were sufficient to prevent efficient replication of ITR5s (Rep25aa116, Rep25aa125, and Rep25aa141). This suggested that these chimeras possessed interruptions of a critical region of Rep5 for ITR5 specificity.

Rep2-based chimeras were unable to replicate ITR5s without the inclusion of the N-terminal 146 aa of Rep5 (Rep52aa146, Fig. 5D). Rep52aa146 replicated ITR5 at wt levels, as did the three chimeras with larger portions of Rep5 on the N-terminus (Rep52aa160, Rep52aa175, Rep52aa207). This mapping reveals that the critical region for ITR specificity in Rep5 lies between aa 97–146. Surprisingly, the Rep52aa146 clone also functioned efficiently on ITR2, constituting a Rep capable of replicating ITR2 and ITR5. This suggested that ITR specificity existed in two different regions of Rep.

For Rep2, the N-terminal 83 or 109 aa of Rep5 could be substituted with no effect on ITR specificity or major influence on replicative fidelity (Rep52aa84 and Rep52aa110, Fig. 5D and 5E). Chimeras including slightly larger portions of Rep5 were unable to replicate either ITR, again suggesting the interruption of a domain critical for ITR specificity (Rep52aa126 and Rep52aa138).

Rep5-based chimeras were unable to replicate ITR2s without the inclusion of the N-terminal 149 aa of Rep2. However, ITR2 replication was inefficient (25aa149, Fig. 5D and 5e). The inclusion of larger portions of Rep2 allowed replication of ITR2s to increase to wt levels (Rep25aa166, Rep25aa216). This data maps the Rep2 region involved in ITR specificity to aa 110–149. However, unlike Rep5, this was not the only region which played a role in ITR specificity. The ability of the Rep52aa146 chimera to replicate ITR2 and ITR5 vectors demonstrated a second region of Rep2 between aa 138–160 sufficient to allow replication of ITR2s even when the other critical region (aa 110–149) was Rep5. The isolation of two different Rep regions involved in ITR specificity was consistent with the discovery of two independent elements governing specificity within the ITR.

### Characterization of Rep Regions Involved in ITR Specificity

**Figure 6:**
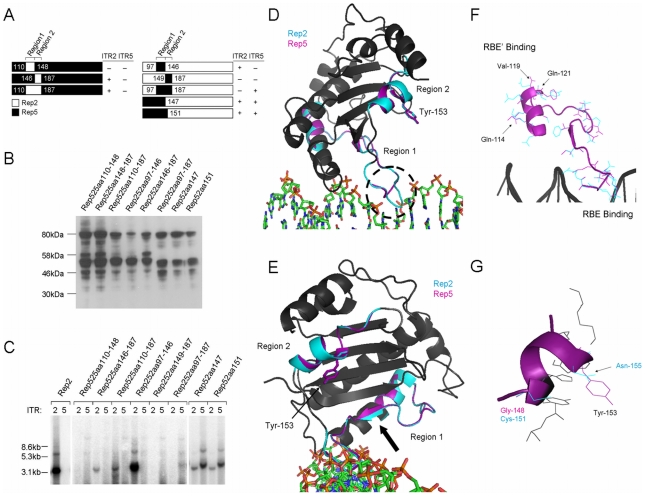
Characterization of Rep regions critical for ITR specificity. (A) Chimeric Reps and their ability to replicate ITR2 or ITR5 flanked vectors. Numbers indicate the aa position of the switch from one Rep to the other. (+) indicates the presence of replication, (−) indicates the absence. Region 1 and 2 involved in Rep-ITR specificity are indicated. (B) Western blot for expression of chimeric Reps. (C) Southern blot demonstrating replication of an ITR2 or ITR5 vector by the chimeric Reps. Note that the ITR5 vector is 500 bp larger than the ITR2 vector. (D) Structural model illustrating the two Rep regions. Rep2 structure is blue, Rep5 is purple. The nucleophilic tyrosine is indicated. Black hatched circle indicates the predicted structural difference of region 1 in the major groove of the ITR. (E) Structural model as in (D). The nucleophilic tyrosine is indicated. (F) Detailed structural view of region 1. The side-chains of non-conserved residues from Rep5 (purple) and Rep2 (blue) are shown. Three Rep5 residues implicated in RBE' binding are indicated. (G) Detailed structural view of region 2. Side chains of active site residues are shown in black. Side chains of non-conserved residues in this region are shown for Rep2 (blue) and Rep5 (purple). The nucleophilic tyrosine is indicated, as is the adjacent Rep2 Asn-155.

To characterize the Rep domains identified in Figure 5, we created chimeric Rep proteins which specifically exchanged the regions implicated in ITR specificity (Fig. 6A). Region 1 existed in Rep2 from aa 110–149 and in Rep5 from aa97–146. Region 2 lay within Rep2 from aa 149–187 and Rep5 from aa 146–187. As in Figure 5, all chimeras were verified by DNA sequencing and Western blot analysis (Fig. 6B). Chimeras were then assayed for the ability to replicate ITR2- or ITR5-flanked vectors (Fig. 6C).

Replacing Rep5 region 1 with Rep2 yielded a clone unable to replicate either vector, suggesting the chimera lacked the ability to bind the ITR5 spacer or nick the ITR2 nicking stem (Rep525aa110–148, Fig. 6C). Replacing Rep5 region 2 with that of Rep2 allowed this chimera to replicate an ITR2 vector, suggesting region 2 of Rep2 was critical to nick the ITR2 nicking stem (Rep525aa146–187). The inability of this chimera to recognize ITR5 is harder to explain as Rep52aa146 could replicate ITR2 and ITR5 efficiently (Fig. 5B). This result suggests that Rep2 region 2 makes specific contacts within Rep2 aa 188–208 which are necessary in order to function on the ITR5 nicking stem. Replacing regions 1 and 2 of Rep5 with Rep2 resulted in a Rep chimera which replicated only ITR2s (Rep525aa110–187).

Replacing Rep2 region 1 with Rep5 resulted in replication of only ITR2s, again demonstrating a connection between Rep2 region 2 and the ITR2 nicking stem (Rep252aa97–146). The lack of ITR5 replication by Rep252aa97–146 is difficult to explain based on the Rep52aa146 chimera which replicates ITR2s and ITR5s efficiently (Fig. 5B). This result suggests that Rep5 region 1 requires specific contacts within the preceding 96 aa of Rep5 in order to replicate ITR5. Replacing Rep2 region 2 with Rep5 resulted in a chimera unable to replicate either ITR (Rep252aa149–187). This chimeric Rep possesses neither Rep2 region 2 (required to nick the ITR2 nicking stem) nor Rep5 region 1 which appears necessary to interact with the ITR5 spacer. Finally, replacing both Rep2 regions 1 and 2 with Rep5 resulted in a chimera capable of replicating only ITR5 vectors (Rep252aa97–187).

The crystal structure of the N-terminal 193 aa of Rep5 complexed to the RBE allowed the location of these two critical regions to be modeled [16]. The structure of the N-terminus of Rep2 was modeled with Swiss-Model software using Rep5 as a template. The location of region 1 supports its involvement with the spacer/RBE (Fig. 6D). This region interacts with the major groove of the ITR where one of the most apparent structural differences between Rep2 and Rep5 is predicted (Fig. 6D, hatched circle). Rep2 contains a two aa insertion in this loop with respect to Rep5. This insertion and other non-conservative substitutions are likely responsible for the inability of Rep2 to interact with the ITR5 spacer.

Viewing Rep along the length of the ITR illustrates that region 1 constitutes much of the base of the protein (Fig. 6E). Both Reps are predicted to participate in a β-sheet motif in the center of this region, while areas of reduced homology exist toward either side (the loop interacting with the major groove of the ITR on one side, RBE' interactions on the other). A more detailed look at region 1 reveals the greatest disparity between Rep2 and Rep5 occurs at the RBE binding interface in the major groove of the ITR (Fig. 6F).

There is very little predicted structural difference between region 2 of Rep2 and Rep5 (Fig. 6D and 6E). In an effort to dissect this region, we created two additional clones: Rep52aa147 and Rep52aa151 (Fig. 6A). Like Rep52aa146, both of these Reps were able to replicate ITR2 and ITR5 vectors (Fig. 6C). Rep52aa146 and Rep52 aa147 replicated ITR2 and ITR5 vectors with equivalent efficiency, suggesting E147 of Rep2 is not involved in ITR specificity. Rep52aa151 did display a modest reduction in ITR2 replication compared to Rep52aa146, suggesting that C151 of Rep2 plays a role in ITR2 specificity. Because Rep52aa160 can not replicate ITR2, this leaves only two other non-conserved residues between Rep2 and Rep5 in this region (N155 and T161). Both of these residues lie near the active site and are likely to interact with the nicking stem or active site. N155 lies directly adjacent to Y156, the nucleophilic tyrosine, and may play a major role in ITR2 specificity (Fig. 6G).

### Structure-Function Model of Rep-ITR Specificity

In order to unify the ITR and Rep elements involved in specificity into a single model, we utilized the chimeric Reps separating region 1 and region 2 along with the chimeric ITRs separating the nicking stem and spacer. Rep2, Rep5, Rep52aa146 (which divides region 1 and 2 of Rep and can replicate ITR2 and ITR5), and Rep25aa149 (essentially no ITR2 or ITR5 replication) were selected. These Reps were tested for their ability to replicate ITR2, ITR5, ITR2+5NS (which is replicated by both Rep2 and Rep5), and ITR5+2NS (which is replicated by neither Rep2 or Rep5).

**Figure 7:**
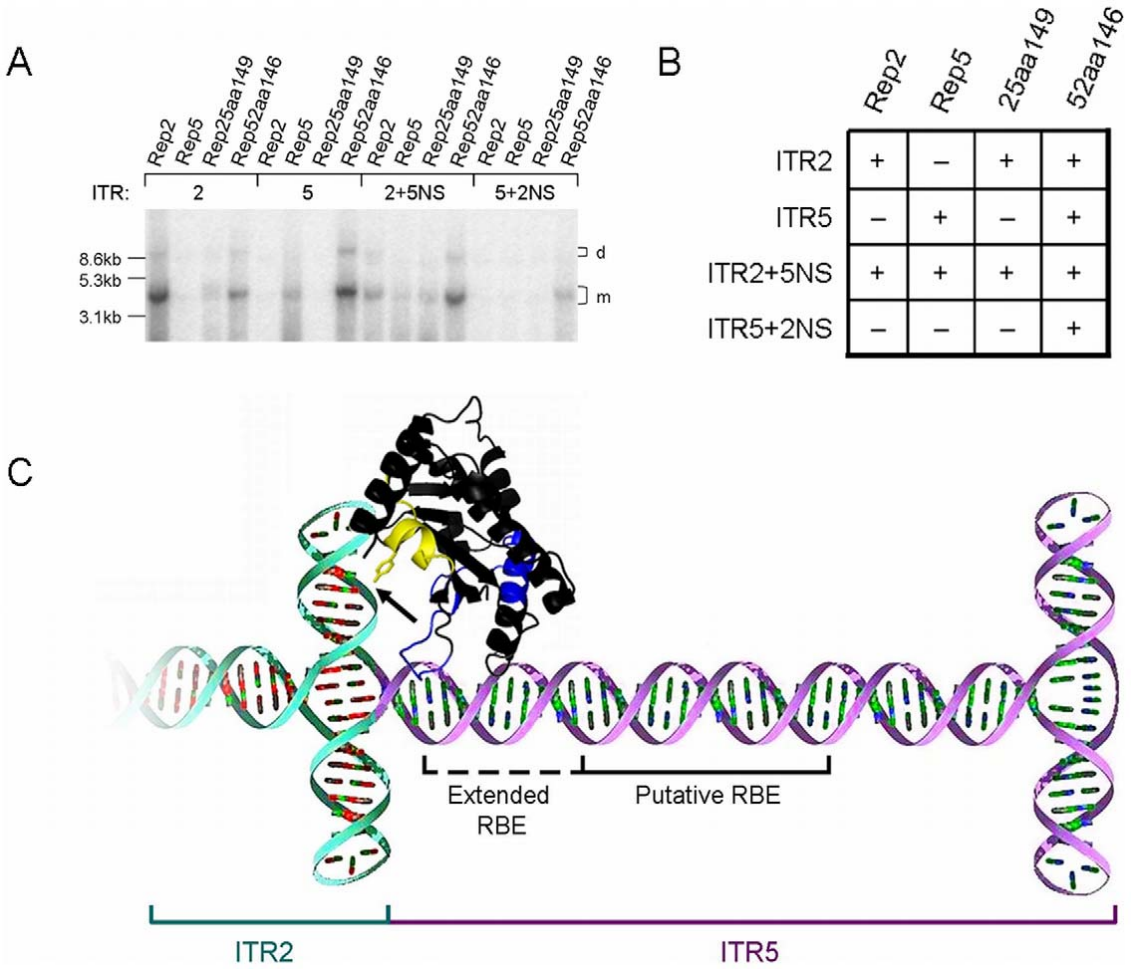
Model of Rep-ITR specificity. (A) Southern blot of Hirt DNA demonstrating replication of the indicated ITR vector by the indicated Rep. (B) Table indicating the presence (+) or absence (−) of replication of the gel from (A). (C) Model of a novel AAV origin of replication. The chimeric ITR can be replicated only by a chimeric Rep protein. Rep5 sequence in region 1 (blue) is required for the extended RBE of ITR5 (purple). Rep2 sequence in region 2 (yellow) is required to function on an ITR2 nicking stem (cyan).

Only Rep2 and Rep52aa146 efficiently replicated ITR2 (Fig. 7A and 7B). Only Rep5 and Rep52aa146 replicated ITR5. As in Figure 1, Rep2 and Rep5 replicated ITR2+5NS. Additionally, Rep25aa149 and Rep52aa146 replicated ITR2+5NS. This ITR appears to be universally replicated by every Rep in this assay due to the exclusion of DNA elements involved in protein specificity. The three nt ITR2 spacer is amenable to the DNA binding region 1 of Rep2 and Rep5. The seven bp tall ITR5 nicking stem functions with region 2 of Rep2 and Rep5. Thus, any combination of these regions constitutes a Rep protein capable of replicating ITR2+5NS.

Finally, neither Rep2 nor Rep5 replicated ITR5+2NS. Rep2 is unable to interact properly with the 15 nt ITR5 spacer. Rep5 is unable to function on the ITR2 nicking stem. For these reasons, Rep25aa149 was also unable to catalyze replication. However, Rep52aa149 was able to replicate this ITR due to the proper combination of Rep regions (Fig. 7C). Rep52aa149 possesses Rep5 region 1 which is necessary to interact with the 15 nt ITR5 spacer. This chimera also possesses Rep2 region 2, essential for function on the ITR2 nicking stem. This recombinant DNA-protein interaction is unique from either AAV2 or AAV5 and constitutes a novel Parvovirus origin of replication.

## Discussion

Taken as a whole, this work illustrates two specific mechanisms of DNA-protein specificity at the Parvovirus origin of replication. Chimeric ITRs narrowed the DNA elements involved in specificity to the spacer and nicking stem sequences (Fig. 1B). These results contradicted previous assertions that Rep-ITR specificity were driven solely by the nicking sequence as Rep2 efficiently nicked an ITR harboring the ITR5 nicking stem [10]. Rep2 is highly flexible in the sequence and height of its nicking stem while Rep5 is highly specific to its cognate stem (Fig. 2).

Three residues of Rep2 are necessary to cleave the ITR2 nicking stem (Fig. 5 and 6). Residues C151, N155, and T161 all lie in the active site of the protein in a predicted alpha helix along with the nucleophilic tyrosine Y156. How these residues (termed Rep region 2) grant Rep2 flexibility toward mutant nicking stems remains unclear. The corresponding Rep5 residues (G148, A152, and V158) may participate in highly specific interactions which require specific height and sequence considerations for the ITR5 nicking stem.

AAV5 Rep-ITR specificity is mediated by the ITR5 spacer. Replacement of the three nt ITR2 spacer with the 15 nt ITR5 spacer ablated replication by Rep2 (Fig. 2B). A poorly conserved Rep binding element allows Rep5 to interact with the elongated ITR5 spacer (Fig. 4B). Mutating the spacer to include a strong Rep binding element allowed Rep2 and Rep5 to replicate the ITR. However, insertion of a Rep binding element into the ITR2 spacer still largely decreased Rep2 function. While this data might suggest that additional Rep5 molecules bind to ITR5, previous *in vitro* experiments have not come to this conclusion, although those studies were performed in the absence of hairpins on the ITRs [10].

A 49 aa region of Rep5 interacts with the ITR5 spacer (aa 97–146, Fig. 5 and 6). The crystal structure of the N-terminus of Rep5 reveals that this region (region 1) possesses residues which specifically bind to the RBE and RBE' of the ITR. Major structural differences in the Rep5 loop which binds the major groove of the RBE likely account for the majority of ITR5 spacer specificity. While Figure 1B predicts RBE' binding should not play a role in Rep-ITR specificity, it is possible that RBE' contacts alter the secondary structure of region 1 as it interacts with the RBE.

Because the regions of Rep critical for ITR specificity were separate (region 1 of Rep5 from aa97–146 and region 2 of Rep2 from aa151–161), a chimeric Rep possessing both regions was able to efficiently replicate ITR2 and ITR5. An ITR which could be replicated by any wt or chimeric Rep was constructed by excluding the DNA elements required for specificity; the ITR5 spacer and the ITR2 nicking stem. Most significantly, a novel origin of replication was generated. This ITR contained both of the critical elements for Rep specificity; the ITR5 spacer and the ITR2 nicking stem. As a result, only a chimeric Rep protein made up of Rep5 region 1 and Rep2 region 2 was able to replicate the ITR. The creation of a unique origin of replication highlights the power of studying the DNA-protein interactions of a viral origin of replication.

The creation of a unique DNA-protein interaction was possible because of the separation of the specific Rep-ITR interactions in AAV2 and AAV5. How and why these two different DNA-protein interactions evolved is unclear. It is likely due to evolutionary divergence in the ITR sequence which may have occurred in different hosts (AAV2 is related to other primate AAVs, AAV5 is related to non-primate AAVs such as goat and bovine). This model of replicative specificity can likely be extended to other parvoviruses such as snake AAV which has a highly conserved T-shaped ITR structure but different spacer and nicking stem lengths [19]. Similar DNA-protein interactions likely occur in distantly related viruses such as the autonomous human Parvovirus B19 which can have ITRs as long as 400 bp [20]. Less conserved Parvovirus origins of replication might also employ additional DNA-protein interactions outside of the nicking stem and spacer sequences.

Additionally, this work may provide further insight into why AAV2 is the only known animal virus capable of integrating site-specifically into the human chromosome [21]. Integration occurs due to the specific cleavage of the AAVS1 site on chromosome 19 by Rep2. Rep2 is highly flexible in its nicking substrates, functioning on nicking stems from five bp to nine bp in height and on poorly conserved trs sequences. Thus, the only requirement for Rep2 to nick the human chromosome would be a functional nicking stem within three nt of a consensus RBE. As there are an estimated 2×10^5^ consensus RBEs in the human chromosome, the likelihood of such an occurrence is high [22]. This may also explain why an integration locus for AAV5 has not been identified. Rep5 is highly specific to both the height and sequence of the ITR5 nicking stem. There is likely no ITR5 nicking stem homolog in the human chromosome within range of a consensus RBE to allow nicking by Rep5. It is possible that other hosts infected by AAV5-related serotypes might possess chromosomal integration sites for AAV5.

These results also stand to improve the safety of future AAV therapeutic vectors. The danger of AAV vector mobilization by wt AAV could be averted if therapeutic vectors harbored ITRs which no wt Rep could replicate [8]. Additionally, the mechanisms of Rep-ITR specificity described here might extend to cellular elements. For instance, the C. elegans mobile element Tc1 contains terminal repeats which are specifically bound and endonucleolytically cleaved by its transposase, Tc1A [23]. Biology at the related SV40 T antigen and papillomavirus E1 origins of replication likely possess conserved interactions [18]. Bacteriophage ϕX174 and plant geminiviruses, as well as other viruses which employ rolling circle mechanisms of replication also possess homology to the AAV origins of replication [24]. In this way, dissection of specificity at the AAV origin of replication provides a broad platform to investigate other DNA-protein interactions.

## Materials and Methods

### Rep Cloning

pXR2 (Rep2Cap2) and pRep5Cap2 AAV helper plasmids served as templates for Rep cloning. The primer sequences used are indicated in Table 1. Two cloning strategies were used. Existing restriction sites were incorporated into primers for PCR (PCR-RD in Table 1) utilizing either pXR out fw or pXR out rev primers. PfuTurbo DNA Polymerase (Stratagene) was used at the manufacturer's recommendations for all PCR reactions. PCR-RD products were digested with the enzymes indicated in Table 1 (NEB) prior to ligation with T4 DNA Ligase (Invitrogen) according to manufacturer's instructions. Alternately, an overlap-extension mediated PCR (OE-PCR) approach was used to produce Rep chimeras [26]. The Rep2 and Rep5 junction was incorporated into forward and reverse primers which were used in separate PCR reactions with the pXR out fw and rev primers (Table 1, only fw oligos indicated, rev oligos complimentary to fw). These overlapping PCR products were combined into a single PCR reaction and cycled as follows: 1 cycle at 94°C for 30 seconds, 18 cycles of 30 seconds at 94°C, 30 seconds at 65°C, and 4 minutes at 72°C, 1 cycle of 10 minutes at 72°C. 1 ul of this reaction was used as template for a nested PCR with the pXR in fw and rev primers. Chimeras with the N-terminus of Rep2 and C-terminus of Rep5 were cloned into the Rep25aa166 construct between the *PpuMI* and *MfeI* sites. Chimeras with the N-terminus of Rep5 and C-terminus of Rep2 were cloned into the 52aa160 construct between the *PpuMI* and *BstBI* sites. All constructs were verified by DNA sequencing at the UNC-CH Genome Analysis Facility.

**Table 1:** Oligonucleotides for chimeric Rep and ITR cloning.

Clone/Primer	Cloning Method	Orientation	Sequence
pXR out fw		Forward	5′ CGAAAAGTGCCACCTGACGTCTAAGAAACC
pXR in fw		Forward	5′ TCGAATTCGACGGCCAGTGAATTGTAATACGACTC
pXR out rev		Reverse	5′ CCATGATTACGCCAAGCTCGGAATTAACCGCATGCGA
pXR in rev		Reverse	5′ CCATGGCCGGGCCCGGATTCACC
Rep52aa84	PCR-RD *AleI*	Reverse	5′ TTCACCCCGGTGGTTTCCACGAGCACGTGCATGTGGAAGTAGCTCTCTCCCTTTTCAAACTGCACAAAG
Rep52aa110	PCR-RD *EagI*	Forward	5′ CCTCGGCCGCTACGTGAGTCAGATTCGCGAAAAACTGATTCAGAG
Rep52aa126	OE PCR	Forward	5′ GTGGTCTTCCAGGGAATTGAACCCACTTTGCCAAACTGGTTCGCGGTC
Rep52aa138	OE PCR	Forward	5′ CTGGGTCGCCATCACCAAGGTAAAGAAGGGAGGCGGGAACAAGGTGGTGGATGAG
Rep52aa146	OE PCR	Forward	5′ GCGGAGCCAATAAGGTGGTGGATGAGTGCTACATCCCCAATTACTTGCTC
Rep52aa160	PCR-RD *Bpu10I*	Reverse	5′ ACTGGAGCTCAGGTTGGACCTTCGGCAGCAGGTAG
Rep52aa175	OE PCR	Forward	5′ CGTGGACAAACCTGGACGAGTATAAATTGGCCTGTTTGAATCTCACGGAGCGTAAAC
Rep52aa187	OE PCR	Forward	5′ CTGAATCTGGAGGAGCGCAAACGGTTGGTGGCGCAGCATCTGACGCAC
Rep52aa207	PCR-RD *SgrAI*	Reverse	5′ GATCACCGGCGCATCCGAGAACTCACGCTGCGAAGC
Rep25aa77	OE PCR	Forward	5′ TAAGGCCCCGGAGGCCCTTTTCTTTGTGCAGTTTGAAAAGGGATCTG
Rep25aa97	OE PCR	Forward	5′ CCACATGCACGTGCTCGTGGAAACCTCCGGCATCTCTTCCATGGTCCTCG
Rep25aa116	PCR-RD *NruI*	Forward	5′ TCAGATTCGCGAAAAACTGGTGAAAGTGGTCTTCCAGG
Rep25aa125	OE PCR	Forward	5′ GAATTTACCGCGGGATCGAGCCG CAGATCAACGACTGGGTCGCCATC
Rep25aa141	OE PCR	Forward	5′ GGTCACAAAGACCAGAAATGGCGCCGGCGGAGCCAATAAGGTGGTGGATTCTGG
Rep25aa149	OE PCR	Forward	5′ GAGGCGGGAACAAGGTGGTGGATTCTGGGTATATTCCCGCCTACCTGC
Rep25aa166	PCR-RD *Bpu10I*	Forward	5′ CCAGCCTGAGCTCCAGTGGGCGTGGACAAACCTG
Rep25aa187	OE PCR	Forward	5′ GTTTGAATCTCACGGAGCGTAAACGGCTCGTCGCGCAGTTTCTGGCAG
Rep25aa216	PCR-RD *SgrAI*	Forward	5′ ATGCGCCGGTGATCAAAAGCAAGACTTCCCAGAAATACATGG
ITR2 Half1 Kpn		Forward	5′ ATTATAGGTACCAGGAACCCCTAGTGATG
ITR2 Half 1 Sfi		Reverse	5′ TAATAGGGCCCAAAGGGCCGGG
ITR2 Half2 Sfi		Forward	5′ TTAATAGGCCCTTTGGGCCGGG
ITR2 Half2 Hind		Reverse	5′ TATAATAAGCTTAGGAACCCCTAGTGATGGAG
ITR5 Half1 Kpn		Forward	5′ ATTATAGGTACCTACAAAACCTCCTTGCTTGAG
ITR5 Half1 Sfi		Reverse	5′ TTAATAGGCCCTTTGGGCCGTCGC
ITR5 Half2 Sfi		Forward	5′ TTAATAGGCCCAAAGGGCCGTCGTC
ITR5 Half2 Hind		Reverse	5′ TATAATAAGCTTTACAAAACCTCCTTGCTTGAGAG

### ITR Cloning

ITRs were cloned into a pUC-18 plasmid with a GFP cassette (CMV promoter, SV40 polyA) cloned between the *KpnI* and *EcoRI* restriction sites. The ITRs were synthesized in two halves as 4nmol Ultramer DNA oligos (Integrated DNA Technologies). *SfiI* restriction sites were incorporated into one hairpin arm the ITR for cloning (Fig. 1A). Due to inconsistencies of the reported sequence at the tip of the ITR5 hairpins between [10], the published genbank sequence (genbank accession number NC_006152), and restriction mapping (data not shown), an ITR2 hairpin was utilized for the ITR5 construct (Fig. 1A). 200pg of each oligo was amplified in a PCR reaction using the ITR primers listed in Table 1. 2.5U of PfuTurbo DNA Polymerase (Stratagene) was used to amplify each half of the ITR as follows: 1 cycle at 94°C for 4 minutes, 35 cycles of 45 seconds at 94°C, 30 seconds at 50°C, and 30 seconds at 72°C, 1 cycle of 10 minutes at 72°C. PCR reactions were purified and subject to digestion by *KpnI* and *SfiI* or *HindIII* and *SfiI* (NEB). A triple ligation with the pUC-18 GFP plasmid and each half of the ITR was performed with T4 DNA Ligase (Invitrogen) for 1.5 hours at room temperature. All constructs were verified by DNA sequencing at the UNC-CH Genome Analysis Facility after linearization of the plasmid and ablation of the ITR secondary structure by *SfiI* digestion.

### Western Blot Analysis

Samples for Western blot analysis were harvested 48–72 hours after transfection of Ad-helper plasmid and the appropriate AAV helper construct. Cells were washed and resuspended in 100 ul PBS prior to addition of 100 ul 2x Laemmli Sample Buffer (100 mM Tris pH 6.8, 4% SDS, 200 mM DTT, 20% glycerol, 0.1% Bromophenol Blue). Samples were briefly sonicated and boiled for 10 minutes. Samples were run on NUPAGE 4–12% Bis-Tris gels (Invitrogen) at 160 volts for 90 minutes. Protein was transferred to a Nitrocellulose membrane (Invitrogen) via a wet transfer for 60 minutes at 30 volts. Gels were blocked overnight in 10% nonfat dry milk in 1× PBS/Tween (0.05%). Detection of both Rep2 and Rep5 proteins (all four sizes) was achieved with a monoclonal Anti-Adeno-Associated Virus Rep Protein antibody (clone 259.5, American Research Products) at a 1∶20 dilution in PBS/Tween for 60 minutes at room temperature. After washing, a secondary HRP anti-mouse antibody was added at a 1∶5,000 dilution in PBS/Tween for one hour at room temperature. After washing, SuperSignal West Femto Maximum Sensitivity Substrate (Pierce) was added and blots were exposed to X-ray film (Kodak).

### Cell Culture and rAAV Production

HEK 293 cells were obtained from ATCC and cultured in Dulbecco Modified Eagle Medium (DMEM) supplemented with 10% Fetal Bovine Serum (Sigma) and 100 units/ml penicillin and 100 g/ml streptomycin and grown at 37°C with 5% CO_2_ saturation. Transfections were performed in six-well cell culture plates. 0.75 ug each of Ad-helper plasmid, AAV helper plasmid (either Rep2Cap2, Rep5Cap2, or the Rep mutant described), and the GFP plasmid containing the ITR (mutant or wt ITR as specified in text) were triple-transfected with PEI (25,000 linear molecular weight) as described [25]. Cells were harvested 48–72 hours post-transfection.

### Hirt DNA Purification and Southern Blot Analysis

Hirt DNA purification was performed as described [27]. Cells were harvested 48–72 hours post-transfection, washed in PBS, and resuspended in 370 ul Hirt Solution (0.01M Tris-HCl pH 7.5 and 0.1M EDTA) prior to addition of 25 ul 10% SDS and 165 ul 5 M NaCl. Samples were incubated at 4°C overnight prior to centrifugation. DNA was purified by phenol chloroform extraction and precipitated by an equal volume of isopropanol prior to resuspension in 50 ul sterile ddH_2_O. 5 ul of each sample was digested with 4U *DpnI* (NEB) 2–4 hours at 37°C prior to gel electrophoresis and Southern blot analysis to remove non-replicated transfected plasmid [28]. The nylon membrane (Hybond-XL, GE Healthcare Life Sciences) was hybridized to a probe corresponding to the GFP open reading frame labeled with the Random Primed DNA Labeling Kit (Roche) and d-CTP P^32^. Blots were visualized after exposure to a phosphorimager screen (GE Healthcare Life Sciences).

### Densitometry

Densitometry was performed using the public domain NIH Image program (developed at the U.S. National Institutes of Health and available on the Internet at http://rsb.info.nih.gov/nih-image/). Densitometry analysis of a *DpnI* resistant band on the agarose gel prior to transfer was used as a loading control to normalize values obtained from the Southern blot. The lowest value (absence of any vector replication) was subtracted from all values to account for background. In order to gauge relative replication efficiency, values for ITR2 vectors were divided by the value obtained from the Rep2-ITR2 control. ITR5 vectors were compared to the Rep5-ITR5 control. All values were obtained in triplicate (n = 3). Error bars represent standard error (standard deviation divided by the root of 3). All samples were compared to controls on the same blot.

### Molecular Modeling

Molecular models were generated using Swiss-Model (http://swissmodel.expasy.org). The published crystal structure of the N-terminus of Rep5 complexed with the RBE (PDB accession #1rz9) was used as a template for all models. Visualization of protein structure rendering of images were performed with PyMOL (http://pymol.sourceforge.net). DNA folding was performed using the DNA mfold server (http://mfold.bioinfo.rpi.edu/cgi-bin/dna-form1.cgil) [29], [30].

## Supporting Information

Figure S1 Illustration of AAV replication. Red indicates newly synthesized DNA. (A) The AAV genome enters the nucleus as a ss DNA molecule flanked by the ds DNA ITRs. (B and C) The free 3′ hydroxyl of the ITR allows second strand synthesis through the opposite ITRs. (D) Rep binds the closed ITR at the RBE and RBE'. The Rep helicase allows the ITR nicking stem to form which Rep cleaves at the trs. (E) Rep remains covalently bound to the 5′ end of the ITR, allowing synthesis through the ITR [31]. (F-G) Complete synthesis of the genome can now occur. (H) The fully replicated genomes can be dissociated by the Rep helicase or by subsequent rounds of DNA synthesis.
(6.48 MB TIF)

Figure S2 Diagram of ITR synthesis. (A) The ITR was synthesized in two pieces (dark blue and light blue) overlapping across one hairpin stem holding the SfiI site (orange). (B) Each half was amplified via PCR prior to digestion and cloning. (C) Proper triple-ligation with pUC18-CMV GFP produced an ITR in DD format.
(2.67 MB TIF)
