# Supplementary figures and images for: Creating a Novel Origin of Replication through Modulating DNA-Protein Interfaces
Source: PLoS One. 2010 Jan 22;5(1):e8850. doi: 10.1371/journal.pone.0008850 (PMC2809752; doi:10.1371/journal.pone.0008850)

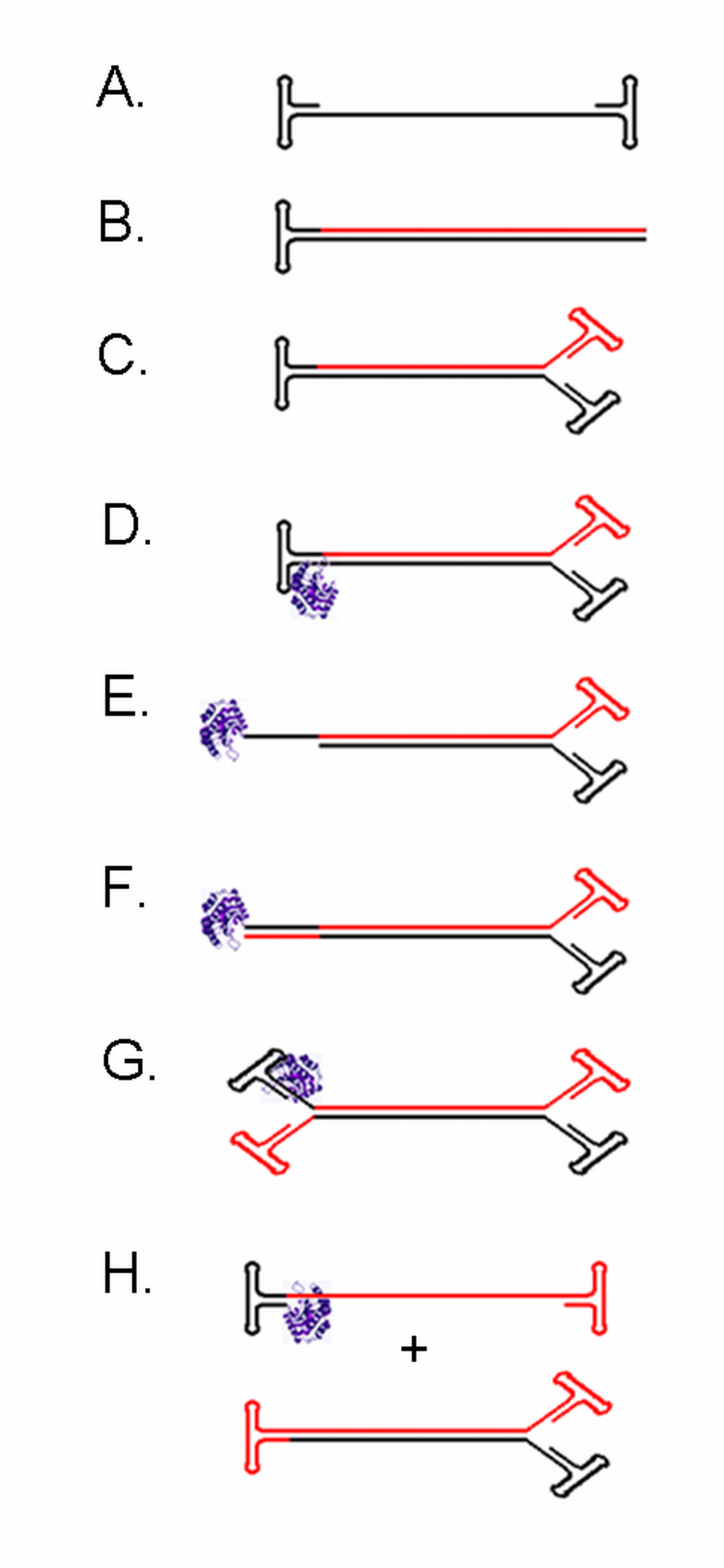

Supplement: Figure S1 — Illustration of AAV replication. Red indicates newly synthesized DNA. (A) The AAV genome enters the nucleus as a ss DNA molecule flanked by the ds DNA ITRs. (B and C) The free 3′ hydroxyl of the ITR allows second strand synthesis through the opposite ITRs. (D) Rep binds the closed ITR at the RBE and RBE'. The Rep helicase allows the ITR nicking stem to form which Rep cleaves at the trs. (E) Rep remains covalently bound to the 5′ end of the ITR, allowing synthesis through the ITR [31]. (F-G) Complete synthesis of the genome can now occur. (H) The fully replicated genomes can be dissociated by the Rep helicase or by subsequent rounds of DNA synthesis. (6.48 MB TIF) [file pone.0008850.s001.tif]

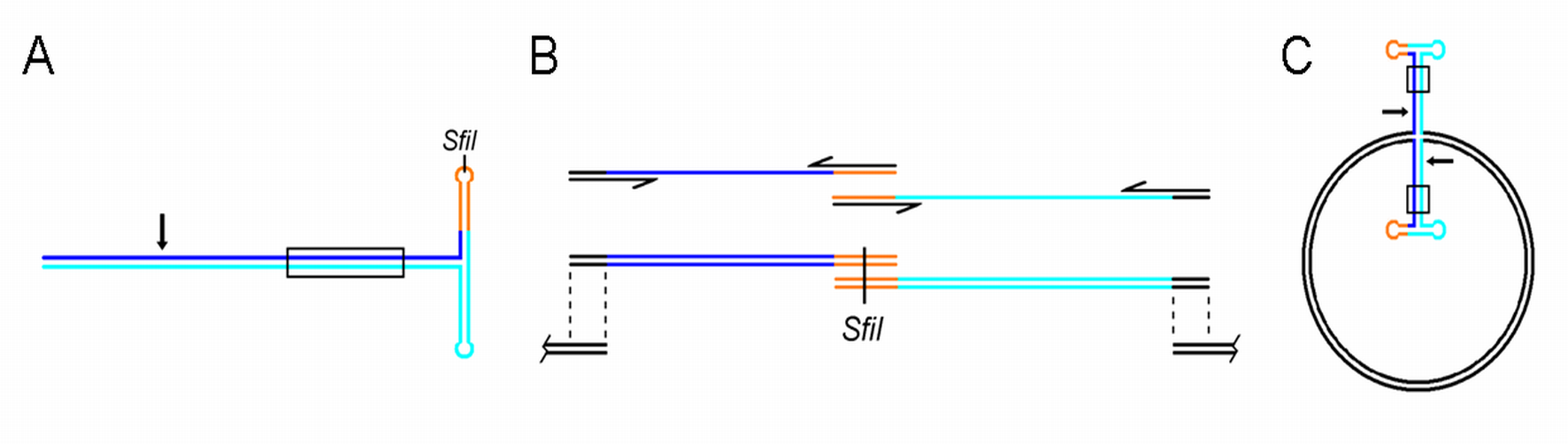

Supplement: Figure S2 — Diagram of ITR synthesis. (A) The ITR was synthesized in two pieces (dark blue and light blue) overlapping across one hairpin stem holding the SfiI site (orange). (B) Each half was amplified via PCR prior to digestion and cloning. (C) Proper triple-ligation with pUC18-CMV GFP produced an ITR in DD format. (2.67 MB TIF) [file pone.0008850.s002.tif]
